# Dysregulation of Mitochondrial in Pulmonary Hypertension-Related Right Ventricular Remodeling: Pathophysiological Features and Targeting Drugs

**DOI:** 10.31083/RCM25781

**Published:** 2025-03-18

**Authors:** Yuehan Wang, Yingzhuo Wang, Weifang Zhang

**Affiliations:** ^1^Departments of Pharmacy, The Second Affiliated Hospital of Nanchang University, 330006 Nanchang, Jiangxi, China; ^2^Huankui Academy, Nanchang University, 330036 Nanchang, Jiangxi, China; ^3^The First Clinical Medical College, Nanchang University, 330036 Nanchang, Jiangxi, China

**Keywords:** mitochondrial dysfunction, pulmonary hypertension, right ventricular remodeling, ROS production, therapeutic targets

## Abstract

Pulmonary hypertension (PH) is a life-threatening condition characterized by right ventricular (RV) remodeling, which is a major determinant of patient survival. The progression of right ventricular remodeling is significantly influenced by mitochondrial dysfunction, providing profound insights into vascular health and cardiovascular risk. In this review, we discuss the molecular targets, pathophysiological characteristics, and potential mechanisms underlying mitochondrial dysfunction in PH, encompassing disturbances in mitochondrial dynamics, inflammation, and dysregulation of mitochondrial energy metabolism. Finally, we review the primary therapeutic targets currently utilized to address cardiac dysfunction resulting from mitochondrial damage. Hopefully, this might inspire novel approaches to the management of cardiovascular disorders.

## 1. Introduction

Pulmonary hypertension (PH) is a rare progressive disorder with high mortality rates, especially among patients over 65 [1]. It is characterized by pulmonary vasoconstriction and vascular remodeling, resulting in increased pulmonary vascular resistance [2]. Current statistics indicate that at least 1% of the global population is affected by PH, with a greater burden more likely in low-income and middle-income countries [3]. This disease remains incurable, substantially impacting patients’ quality of life and posing a threat to their survival. Despite the initial involvement of the pulmonary vasculature in PH, the right ventricle (RV) plays a crucial role in determining clinical outcomes [4]. Studies have shown that right heart failure is a leading cause of morbidity and mortality in PH [5]. In hospitalized patients with pulmonary arterial hypertension (PAH), the mortality rates from right heart failure exceed 40% [6]. Initially, right ventricular hypertrophy (RVH) triggered by pressure overload is a compensatory mechanism, where the RV adapts to increased afterload by thickening its walls and enhancing contractility. However, as pulmonary artery pressure escalates, the right heart transitions from a compensated to a decompensated state, ultimately leading to right heart failure [7]. Pulmonary hypertension induces right ventricular remodeling through various mechanisms, including the enlargement of myocardial cells, promotion of myocardial fibrosis, and alterations in energy metabolism [8].

Mitochondria serve as the primary sites for adenosine triphosphate (ATP) synthesis and have a substantial impact in regulating reactive oxygen species (ROS) production, mitochondrial biogenesis, fusion and fission, mitosis, and calcium homeostasis [9]. They also play a pivotal role in right heart remodeling. In an experimental model of PH induced by hypoxia, the Warburg metabolic phenomenon is evident, characterized by diminished oxidative phosphorylation and increased glycolysis [10]. This altered metabolic pattern results in irregular mitochondrial function. On the one hand, mitochondria dysfunction leads to energy metabolism disorders in pulmonary artery smooth muscle cells (PASMCs), and a reduction in ATP production. As pulmonary hypertension progresses, the demand for ATP in the RV increases, creating a state where ATP supply fails to meet demand. On the other hand, this dysfunction generates excessive ROS, heightens oxidative stress, and activates inflammatory responses [2], accelerating the progression of right heart failure.

This article reviews the mechanisms of mitochondrial changes induced by PH that leads to ventricular remodeling. In the meanwhile, emerging targeted drugs, new therapeutic targets, and emerging technologies all manifested potential in protecting myocardial cells from damage and alleviating ventricular remodeling and cardiac dysfunction. However, we tried to summarize those strategies aimed at addressing mitochondrial dysfunction in PH patients.

## 2. Mitochondrial Dysfunction in Right Ventricular Remodeling after PH

Mitochondria are the primary sites of intracellular material metabolism and energy metabolism. Mitochondrial dysfunction is ubiquitous in cardiovascular diseases [11], involving processes such as metabolic dysregulation and kinetic abnormalities. In the past few decades, a growing array of studies have suggested that mitochondrial dysfunction is intimately linked to RV remodeling brought on by pulmonary hypertension. The precise molecular pathways and clinical presentations of mitochondrial dysfunction will be outlined here.

### 2.1 Dysregulation of Mitochondrial Energy Metabolism

Mitochondria serve as the major sites for oxidative phosphorylation within the cell, providing a continuous supply of energy required for the regular progression of living activities. Mitochondria have an especially important role in tissues and organs with high energy demands, like the heart.

**Fig. 1.**
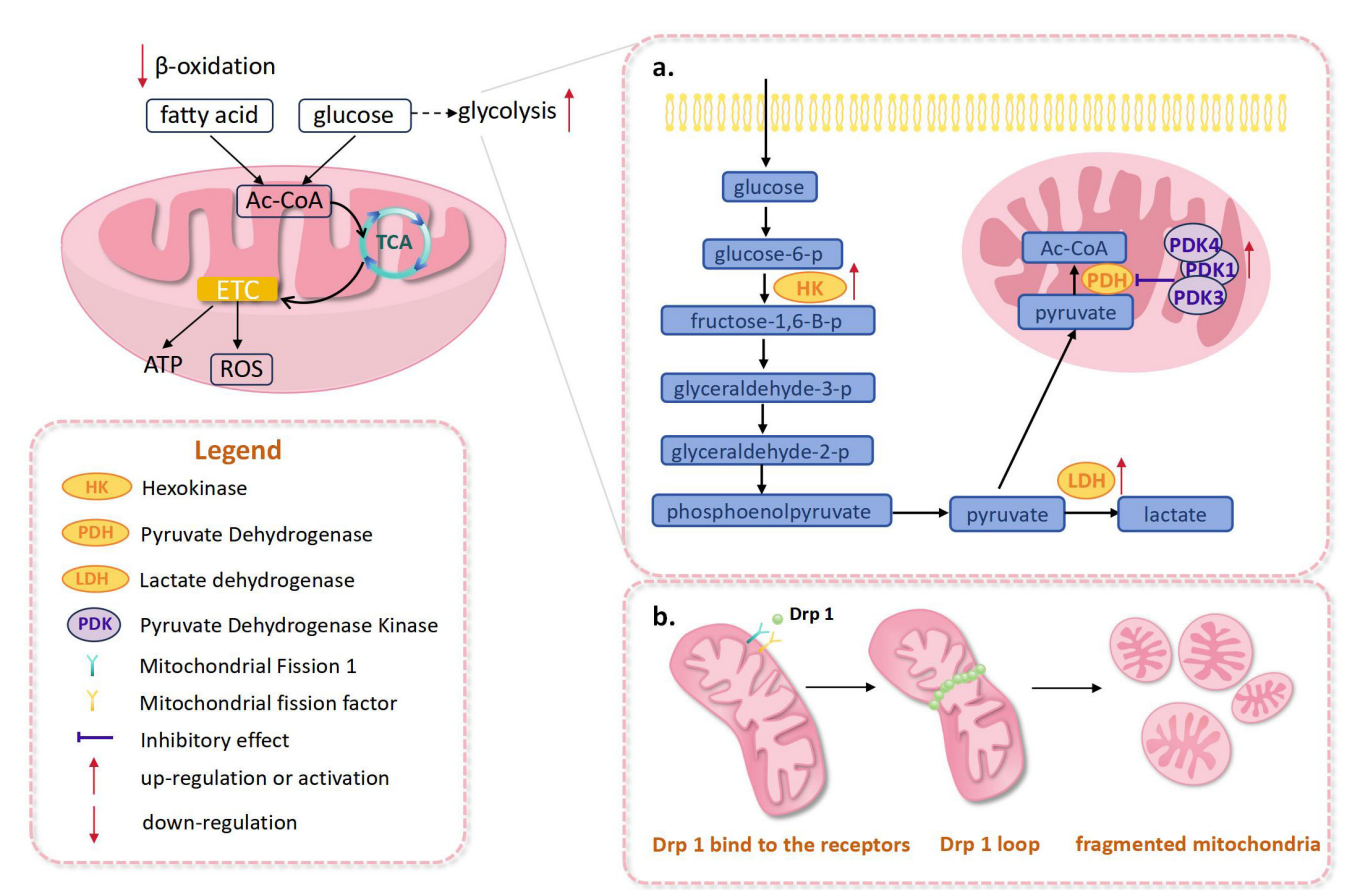
**Abnormal changes in the mitochondria of right heart after pulmonary hypertension**. (a) In right heart pulmonary hypertension, activation of pyruvate dehydrogenase kinase, lactate dehydrogenase, and hexokinase were observed, which promoted the enhancement of glycolysis. (b) Drp1-mediated mitochondrial fission enhanced, leading to the increased fragmentation of mitochondria. ETC, electron transport chain; ROS, reactive oxygen species; Drp1, dynamin-related protein 1; TCA, tricarboxylic acid cycle; Ac-CoA, acetyl-coenzyme A; ATP, adenosine triphosphate.

Under normal physiological conditions, the main source of energy for the heart is fatty acid β-oxidation [12]. Cardiac load, oxygen supply, and hormone levels may affect substrate utilization in cardiac cells [13]. In pulmonary hypertension-induced right heart hypertrophy, myocardial fatty acid uptake is impaired [14]. During heart failure, the major energy production pathway in cardiomyocytes switches from mitochondrial fatty acid oxidation to glycolysis, in order to sustain ATP levels [15]. Increased glycolysis and decreased mitochondrial oxidation may be metabolic indicators of ventricular remodeling after pulmonary hypertension [16] (Fig. 1).

One of the main causes of mitochondrial metabolic dysregulation in PAH is activation of pyruvate dehydrogenase kinase (PDK), which inhibits pyruvate dehydrogenase (PDH) [17] (Fig. 1a). In a PAH model, right ventricular fibroblasts (RVfib) exhibit PDK-dependent reprogramming of mitochondrial metabolism. In particular, upregulated PDK1 and PDK3 led to enhanced mitochondrial fragmentation as well as increased collagen production, which promote right ventricular fibrosis [18]. In a previous study, Piao *et al*. [19] used mitochondrial electron transport chain (ETC) complex 1-deficient Fawn-Hooded rats (FHR), to confirm that RVH in PAH is associated with the switch from glucose oxidation to glycolysis. The results suggested that upregulation of forkhead box protein O1 (FOXO1) could lead to a higher level of PDK4, which in turn inhibited PDH activity, reduced glucose oxidation and impaired right ventricular function. Hexokinase is a critical enzyme in glycolysis. Hexokinase activity was significantly increased in the failed right ventricle, compared with the compensated hypertrophied right ventricle (Fig. 1a). However, lactate dehydrogenase activity was increased only in compensated hypertrophied right ventricles (Fig. 1a). These changes in metabolic enzymes may be associated with increased mitochondrial oxygenation during right heart failure [20]. In another study, pulmonary hypertension was found to induce an increase in the ratio of pyruvate kinase isozyme typeM2 (PKM 2)/pyruvate kinase isozyme typeM1 (PKM 1) in bilateral ventricles of rats [21].

### 2.2 Oxidative Stress in Mitochondria

Mitochondria are the primary site of ROS production in cardiomyocytes. There are several sources of ROS in mitochondria, including ETC complexes and mitochondrial proteins [22, 23]. Under normal conditions, intracellular levels of ROS remain relatively stable due to the presence of antioxidant systems. However, it has been suggested that abnormal ROS production may be associated with maladaptive remodeling of the right ventricle.

Methylation-controlled J protein (MCJ) is a transmembrane protein in the mitochondrial inner membrane, which regulates mitochondrial metabolism by modulating complex I activity and oxidative stress [24]. Santamans *et al*. [25] found that MCJ expression was increased in cardiomyocytes after pulmonary hypertension. MCJ knockout could lead to the suppression of right heart hypertrophy and significant improvement in right heart function, accompanied by elevated ROS levels. Mechanistic studies indicated that MCJ may exert a protective effect by activating the ROS/mammalian target of rapamycin (mTOR)/hypoxia-inducible factor 1-α (HIF1-α) axis. Monoamine oxidase (MAO), located in the outer mitochondrial membrane, is closely associated with ROS production [26]. Specific knockout of cardiomyocyte monoamine oxidase B resulted in a reduction of ROS in right heart tissue, a prominent reduction in the activity of members of the rapidly accelerated fibrosarcoma (RAF) kinase family (A-Raf proto-oncogene, serine/threonine kinase (ARAF)/B-Raf proto-oncogene, serine/threonine kinase (BRAF)/Raf-1 proto-oncogene, serine/threonine kinase (RAF1)), and significant amelioration of RV hypertrophy as well as dilatation [27]. In addition, uncoupling protein 2 (UCP2) is an important mitochondrial inner membrane transporter protein that regulates ROS production [28]. Nevertheless, compared with the wild type, UCP2 deletion resulted in decreased proliferation and increased collagen content in mouse cardiac fibroblasts, but no significant changes in ROS. A certain degree of fibrosis may have a protective effect on the right heart after PAH, avoiding the transition of the RV to a maladaptive type of hypertrophy [29]. Overall, in PH, mitochondrial ROS probably acts as a signaling molecule that regulates changes in right ventricular function.

### 2.3 Disturbances in Mitochondrial Dynamics

The continuous process of mitochondrial division and fusion is a highly plastic regulatory network influenced by multiple regulatory factors. It is reported that mitochondrial dynamics serve as a key link in the mitochondrial quality control mechanism [30]. This dynamic balance can regulate intracellular mitochondrial morphological heterogeneity, which can influence biological processes such as energy production and maintenance of organelle integrity [31]. Abnormal activation of mitochondrial division leads to increased mitochondrial fragmentation, which is a characteristic frequently observed in cardiovascular disorders [32, 33].

Mitochondrial division is largely dependent on the activation of dynamin-related protein 1 (Drp1), an important division factor [34]. When stimulated by signals from inside and outside the cell, Drp1 will be activated by phosphorylation or dephosphorylation modifications at the serine 637 and serine 616 sites [35]. Consequently, a Drp1 loop on the outer mitochondrial membrane was formed to induce membrane contraction and mitochondrial fragmentation [36]. In RV myocytes from PAH patients, excessive mitochondrial fragmentation can lead to increased mitochondrial ROS production, which is associated with impaired RV diastolic function [37]. In addition, in monocrotaline (MCT)-induced PAH rats, mitochondrial fragmentation, RVfib activation, and overproduction of type III collagen were observed. Overall, hyperactivation of Drp1 may be the underlying mechanism [38] (Fig. 1b).

Mitochondrial fusion is coordinately mediated primarily by the factors mitofusin 1 (Mfn1), mitofusin 2 (Mfn2), and optic atrophy-1 (Opa1). Mfn1 and Mfn2 are localized to the outer mitochondrial membrane [39] and mediate the bolus and fusion of the outer membrane [40]. Opa1 is localized to the inner mitochondrial membrane, responsible for the fusion of the inner mitochondrial membrane, and is involved in cristae shaping [41]. Using a rat model of PH induced by Su 5416 combined with hypoxia (SuHx) treatment, Luo *et al*. [42] demonstrated an important role for the mitochondrial membrane fusion factors Opa1 or Mfn2 in myocardial hypertrophy and compensated RV remodeling. In SuHx rat RV tissues, the expression of mitochondrial fusion proteins Opa 1 and Mfn2 was significantly downregulated, whereas the expression of cardiac hypertrophy-related genes (e.g., *Nppb* and *Myh 7*) was significantly upregulated. In PH rat RV tissues, the number of mitochondria was increased, accompanied by a decrease in mitochondrial circumference and area. In the hypoxic cardiac hypertrophic cell model, mitochondrial fusion was reduced and division increased, accompanied by a significant increase in ROS production, an alteration that was reversed by overexpression of Opa 1 or Mfn2. This evidence suggests that the mitochondrial fusion factors Opa 1 or Mfn2 may be involved in the development of maladaptive RV remodeling by regulating mitochondrial function ROS production.

### 2.4 Inflammation and Mitochondrial Damage

In addition to energy metabolism disorders and the disturbance of mitochondrial dynamics, a recent study focusing on the function activity of macrophages, provides a new idea for the research on RV function damage after PAH. In this study, Al-Qazazi R *et al*. [43], using SuHx or wild larkspurine-induced PH rat models, found that the inflammatory response, instead of increased afterload, induced by macrophage mitochondrial dysfunction is a key factor leading to myocardial fibrosis and decompensated RV remodeling.

## 3. Mitochondrial Therapeutic Targets in Right Ventricular Remodeling after PH

As previously stated, mitochondria play an important role in PH, especially in pulmonary vascular and right ventricular remodeling. Therefore, addressing this specific phenomenon could be an important therapy [44]. Enhancing mitochondrial oxidative phosphorylation (OXPHOS), suppressing glycolysis, correcting mitochondrial imbalance, inhibiting mitochondrial fission, and promoting autophagy are all promising therapeutic targets.

### 3.1 Targets Based on Mitochondrial Metabolism

The primary role of mitochondria is to generate ATP through OXPHOS, but when people have PH, their mitochondrial metabolism shifts towards glycolysis. Decreasing glycolysis and reinstating OXPHOS may represent an effective therapeutic approach.

#### 3.1.1 Fatty acid oxidation (FAO) Inhibitors

FAO is the major source of ATP production in the adult heart whereas glucose metabolism is considered a secondary source [45]. There exists a reciprocal relationship between two principal oxidative metabolic pathways, such that the inhibition of FAO increases glucose [46]. This phenomenon is referred to as the Randle cycle [47]. Trimetazidine and ranolazine are two long-chain FAO inhibitors that improve RV function and indirectly activate PDH [48, 49].

Trimetazidine (TMZ) is a partial inhibitor of lipid oxidation and has cardioprotective effects without affecting heart rate, blood pressure, or concurrent therapies [50]. Its mechanism of action is closely linked to mitochondrial metabolism. Kuzmicic *et al*. [51] revealed that TMZ at low concentrations improved mitochondrial function by potentiating all the metabolic parameters assessed. They treated cultured rat cardiomyocytes with TMZ and evaluated lipid accumulation by confocal fluorescence microscopy and parameters of mitochondrial metabolism, finding an increase in lipid accumulation, mitochondrial membrane potential, oxygen consumption rate (OCR) and ATP levels. These metabolic parameters suggest that TMZ is able to increase lipid accumulation and promote mitochondrial metabolism in myocardial cells. Furthermore, TMZ has been shown to protect cardiomyocytes from palmitate-induced mitochondrial fission and dysfunction, without altering the expression levels of several proteins associated with mitochondrial dynamics.

Hypoxic pulmonary hypertension (HPH) is a progressive disease characterized by hyper-proliferation of pulmonary vascular cells including PASMCs, which can ultimately lead to right heart failure and premature mortality [52]. Subsequent research incubated human PASMCs with TMZ prior to hypoxic exposure, restoring mitochondrial potential and respiratory rates, which indicated that TMZ exerted a protective role against hypoxia-induced PASMC proliferation by preserving mitochondrial function [53]. Beyond its mitochondrial benefits in PH, TMZ also exhibits other functions such as an indirect antioxidant effect: a short course of TMZ increases antioxidant activity and decreases oxidative stress [54, 55]. It has been shown that a short course of TMZ is safe and well-tolerated on PAH patients, warranting further evaluation of its therapeutic potential in larger clinical trials.

Another FAO inhibitor, ranolazine, appears to have a similar benefit in terms of outcomes on RV function and exercise capacity in PAH patients. Khan *et al*. [56] enrolled 11 patients to evaluate the safety and efficacy of ranolazine in patients with PH. Except 1 patient who experienced a drug-drug interaction after 3 days of therapy, 8 (80%) of 10 completed all study tests. 3 months later, it appeared that ranolazine had led to an improvement in functional class (*p* = 0.0013), reduction in RV size (*p* = 0.015), and improved RV function (*p* = 0.037) which indicated it could improve symptoms and echocardiographic parameters of RV structure and function safely and effectively. However, it is worth noting that ranolazine was not associated with an improvement in invasive hemodynamic parameters, suggesting that its main role is to improve myocardial metabolism.

#### 3.1.2 mTOR Inhibitors

The mTOR is a master regulator of cell growth, proliferation, and survival [57]. The mTOR pathway comprises two distinct complexes: growth-promoting mTOR complex 1 (mTORC1) and pro-survival mTOR complex 2 (mTORC2). These complexes exhibit varying patterns within the pulmonary vasculature and RV in rats with SU5416/hypoxia-induced PH. mTORC1 signaling accompanied by cardiomyocyte and RV hypertrophy, increased RV wall thickness (RV WT) which is closely related to the process of RV remodeling. Research also shows that mTOR inhibitors reverse RV remodeling and improve RV structure and function in rats, highlighting their potential as therapeutic targets [58].

In the context of HPH, the activation of HIF1-α results in the upregulation of glycolytic genes and mitochondrial fission. The former reprogramming diminishes FAO, contributing to lipid accumulation in cardiomyocytes and subsequent RV remodeling and dysfunction [59, 60]. For the latter, the activation of HIF1-α results in mitochondrial fission via the phosphorylation of Drp1 serine 616 [36]. By targeting HIF1-α, mTOR inhibitors such as rapamycin or everolimus also act on pulmonary vascular proliferation, especially for patients with HPH [61, 62, 63].

Rapamycin is a potent anti-proliferative drug that exerts action by inhibiting its target, mTOR [64]. It effectively inhibits both mTORC1 and mTORC2. mTORC1 acts as a metabolic regulator. Its activation enhances glycolysis while inhibiting autophagy. By inhibiting mTORC1, rapamycin reduces the pathological hypertrophy of cardiac myocytes and improves cardiac function [58]. Recent findings suggest that, in response to certain hypertrophic stimuli, signaling via mTOR is required for the activation of protein synthesis and cardiac hypertrophy [65]. Rapamycin inhibits mTOR and then blocks mitogen-induced signaling via phosphoinositide 3-kinase (PI3K) and protein kinase B (Akt) to the cell cycle machinery in smooth muscle cells (SMCs) *in vitro* and *in vivo* [66], and then inhibits mTOR by the PI3K-Akt-mTOR signaling pathway. It is important to note that varying doses of rapamycin yield different effects on Akt activity. Low concentrations of mTORC1 inhibition can enhance Akt protein activity, whereas high concentrations produce the opposite effect, with the mTORC2 pathway becoming predominant [67]. Rapamycin not only prevents but also reverses vascular remodeling processes and RV signs of PH in mice held under hypoxic conditions [61]. However, it reminds us it is imperative to find a state of equilibrium as soon as possible, as rapamycin is located at the optimal concentration for treating ventricular remodeling after hypertension.

While rapamycin is the first generation of mTOR inhibitors, mTOR kinase inhibitors are the second generation. mTOR kinase inhibitors exploit the structure of the ATP binding pocket of the kinases with small molecules that compete for the binding pocket with ATP, so they are called ATP competitive inhibitors [68]. Studies show that ATP-competitive inhibitors improve the metabolomic profile of microvascular pulmonary arterial vascular smooth muscle cells (PAVSMCs) from subjects with PAH [69], selectively reducing proliferation and promoting apoptosis in human PH, reversing hypoxia-induced pulmonary vascular remodeling and RV remodeling, improving right ventricle structure and function in rats [58, 70].

Nowadays there are other inhibitors such as PI3K/mTOR dual inhibitors which are more effective in preventing or reversing the process of ventricular remodeling by simultaneously inhibiting two key signaling pathways, PI3K and mTOR. However, these are associated with side effects such as high blood sugar, rash and diarrhea, indicating that further research is needed [71].

#### 3.1.3 PDK Inhibitors

Dichloroacetate (DCA) is a typical PDK inhibitor of mitochondria and has been shown to reverse RV remodeling, restore mitochondrial function, and regulate mitochondria-dependent apoptosis [72]. The first clinical trial using DCA demonstrated a significant reduction in mean pulmonary artery pressure (PAP) and pulmonary vascular resistance (PVR) in patients with genetic susceptibility to PAH, confirming the interest of PDK as a therapeutic target [73]. However, there are some important points to note: (1) DCA is restricted to the hypertrophied RV. In *ex vivo* perfused hearts, findings supported that DCA did not have significant effects on the normal RV, but rather on RVH; (2) some patients with functional variants of sirtuin 3 (SIRT3) and UCP2 that predict reduced protein activity did not respond to DCA, suggesting that PDH inhibition in these patients was less PDK dependent [74]. This underscores the importance of personalized medicine and precision medicine; (3) DCA may have an effect on activated immune cells in PAH, but further studies are needed to explore this pathway [75].

Nowadays more and more clinical evidence reveals that DCA has the ability for a combined ‘double-hit’ mechanism, wherein pulmonary vascular remodeling and PAH are reversed and at the same time RV function is directly enhanced, which is very desirable clinically [76]. Investigating its molecular mechanism, DCA is a competitor of pyruvate-PDK binding which can reverse the Warburg effect in pulmonary arterial SMCs and RV cardiomyocytes, reduce glycolysis, and restore OXPHOS metabolism, ultimately leading to PAH improvement and RV remodeling [17, 53, 77].

Currently, there is a growing body of research focused on the synergistic potential of combining DCA with other pharmacological agents, such as tyrosine kinase inhibitors, given that tyrosine kinases can inhibit both PDH and active PDK [78]. Although drug combination therapy requires careful consideration of drug-drug interactions and potential side effects to ensure the safety of treatment, it could be worthwhile to anticipate this outcome.

#### 3.1.4 Traditional Chinese Medicine

In recent years, researchers have found that herbal formulas have certain advantages in the treatment and prevention of PH, and modern pharmacological studies have shown that a variety of herbal formulas can effectively alleviate the symptoms of pulmonary hypertension and right heart remodeling in preclinical settings [79].

Qiliqiangxin (QLQX), a traditional Chinese medicine, can assist in clearing meridian blockage during the progression of heart failure by enhancing blood circulation [80]. It has recently been reported that QLQX combined with targeted drugs significantly attenuated RV remodeling in addition to lowering pulmonary artery pressure. Lu *et al*. [81] revealed that QLQX effectively diminished the mitochondria-associated apoptotic pathway and reversed mitochondria-related metabolic shift. In *in vitro* experiments on mice, QLQX treatment significantly increased superoxide dismutase 2 (SOD2) and decreased cytochrome c, which was beneficial for restoring mitochondrial function. All these processes help to inhibit PAH-induced RV remodeling [81]. There is considerable experience with QLQX in patients with heart failure, with reports of favorable effects on symptoms, functional capacity, and outcomes. Sun *et al*. [80] presented a summary of 129 randomized controlled trials that evaluated QLQX, involving 11,547 patients with heart failure, assignment to QLQX was accompanied by a 51% reduction in hospitalization for heart failure and a 47% reduction in all-cause mortality. Furthermore, QLQX was found to lower the combined risk of cardiovascular death or heart failure hospitalization in numerous investigations [82].

Qibaiping capsules have been found to down-regulate the expression of Bax, cytochrome (CytC), Caspase-9, and Caspase-3 in oxygen-induced PASMCs of rats, while also decreasing B-cell lymphoma-2 (BCL2) expression, effectively modulating the mitochondrial apoptosis pathway, promoting PASMC apoptosis, and improving pulmonary vascular remodeling [83]. Other traditional Chinese medicines like Astragalus injection could also significantly improve the right ventricular function in PAH model rats [84].

### 3.2 Targets Based on Mitochondrial Dynamics

Mitochondria are dynamic organelles that undergo cycles of fission and fusion [85]. In PAH, altered mitochondrial dynamics (i.e., increased division–named fission–and decreased fusion) would promote the proliferation/resistance to apoptosis phenotype in pulmonary arterial cells [74]. Increased cytoplasmic GTPase Drp1 in pulmonary arterial SMCs, peroxisome proliferator-activated receptor gamma coactivator-1 alpha (PGC1 α), and HIF1-α are involved in this mechanism [53].

#### 3.2.1 Drp1 Inhibitors

Drp1, the key molecule in mitochondrial fission, mediates mitochondrial fission while also affecting mitochondrial fusion and autophagy through numerous pathways [86]. Numerous clinical studies have shown that the downregulation of Drp1 with inhibitors can reverse PAH [38, 53, 86]. The phosphorylation of serine sites 637 and 656 lowers Drp1 activity and then prevents mitochondrial fission, whereas the phosphorylation of serines 616, 579 or 600 causes mitochondrial fission by enhancing Drp1 activity [87, 88, 89, 90]. Drp1 inhibitors such as mitochondrial division inhibitor 1 (Mdivi-1) and small interfering RNA Drp1 (siDrp1) prevented mitochondrial fragmentation and reduced the proliferation of SMCs [36].

Mdivi-1 is a quinazolinone derivative that is widely reported to inhibit Drp1-dependent fission, elongate mitochondria, and mitigate brain injury [91]. Tian *et al*. [38] evaluated mitochondrial fission in RVfib from rats with MCT-induced PAH and set up an experimental group intervened with Mdivi-1. They concluded that Mdivi-1 inhibited mitochondrial fission, proliferation and collagen III expression in MCT-RVfib. Despite being the most widely used preclinical Drp1 inhibitor, Mdivi-1 has numerous concerns including its proposed off-target effects which report a lack of cytoprotective effects and increased cell death with Mdivi-1 [92].

Researchers recently found an alternative Drp1 inhibitor which bound directly to Drp1——Drp1i27. In cell line models, a dose-dependent response was observed in Drp1 wild type mouse embryonic fibroblasts (MEFs), but had no significant effect on Drp1 knockout (KO) MEFs, indicating a DRP1-dependent effect. They found that Drp1i27 targets Drp1-mediated mitochondrial fission in cell line models and protects against simulated ischemia-reperfusion injury. Although the study provided promising *in vitro* and *in vivo* data, Drp1i27 has not been evaluated in clinical trials to date [93].

#### 3.2.2 LCZ696 (Sacubitril/Valsartan) 

LCZ696 (sacubitril/valsartan) is an angiotensin receptor-neprilysin inhibitor drug, consisting of a 1:1 mixture of the neprilysin inhibitor sacubitril and the angiotensin receptor blocker valsartan [94]. LCZ696 has been demonstrated to promote left ventricular (LV) reverse remodeling and improve outcomes in patients with heart failure with reduced ejection fraction [94]. Researchers used Sprague–Dawley rats through banding of the main pulmonary artery to evaluate the effects of LCZ696 treatment on the biomechanical properties of failing RV myocardium. Then they found a dual mechanism of action whereby LCZ696 was able to efficiently manage blood pressure while also improving ventricular remodeling and inhibiting myocardial hypertrophy [95]. According to Shen *et al*. [96], LCZ696 attenuated RV remodeling by downregulating PDK4 and inhibiting PDK4/phosphorylation of glycogen synthase kinase-3β (p-GSK3β). In adult C57 mice subjected to pulmonary artery constriction (PAC), mice treated with LCZ696 exhibited notably diminished RV wall thickness and diameters, reduced myocardial fibrosis, decreased expression of PDK4 protein, and reduced p-GSK3β.

Xia *et al*. [97] indicated that LCZ696 could improve cardiac function and reduce apoptosis partly by preserving mitochondrial function via the Drp1-mediated pathway. There is also clinical evidence that an increase in Drp1 expression can be observed in human failing hearts, which favors the transition to mitochondrial fission. Although there are few studies on the effect of mitochondria in pulmonary hypertension, some researchers have reported LCZ696 to reduce mitochondrial ROS levels and preserve mitochondrial integrity, which provides a new research direction [98]. An increasing amount of evidence indicates that LCZ696 is safe and well tolerated, meaning this novel and orally bioavailable drug has potential for further clinical development [99].

### 3.3 Potential Therapeutic Targets and New Therapeutic Techniques

As noted, we have introduced two main targets based on mitochondrial metabolism and dynamics. The former plays a major role in the metabolic transition from glycolysis to OXPHOS. The latter mainly acted on promoting mitochondrial fusion and inhibiting division. Nevertheless, there are other potential therapeutic targets with prospects in the process of RV remodeling induced by PH, although there haven’t been many clinical trials.

#### 3.3.1 Electron Transport Chain

The ETC is located in the inner membrane of the mitochondria and consists of a series of five complexes (complexes I, II, III, IV, and V, the ATP synthase), mitochondrial ATP generation and ROS production are intimately linked through the function of the ETC [100]. Thus, targeting ETC complexes could modulate overall mitochondrial metabolism.

In rat models, studies suggest ETC abnormalities in PAH [74]. The disturbance in the functioning of the ETC complexes induces a disturbance in the production of ROS, mainly because of a decrease in the expression and activity of complex II (subunit B), consequently, they can inhibit the development of PAH-induced RV heart failure [101]. However, these discoveries are preliminary and need to be confirmed by further studies, but specific targeting of ETC complexes could be an interesting therapeutic option.

#### 3.3.2 Mitochondrial Transplantation

Mitochondrial dysfunction is a key factor in the progression of PH [102]. Recent studies have investigated the potential of mitochondrial transplantation in experimental PH. In the monocrotaline model of PH, mitochondria were extracted from the soleus muscles of rats and transplanted into the same rats via intravenous delivery for distribution to the systemic circulation. Here, mitochondrial transplantation restored pulmonary artery vasoreactivity, increased lung tissue adenosine triphosphate concentrations, and reduced the afterload and RV remodeling in rats [103]. What is exciting is that mitochondrial transplantation did not alter the survival of these animals. In the experimental rats with hypoxia-induced pulmonary hypertension, the intact mitochondria were transplanted from femoral artery SMCs into pulmonary artery SMCs *in vivo* via intravenous administration, and interestingly, they displayed reduced vasoconstriction and less pulmonary vascular remodeling [104]. Although mitochondrial transplantation is a fairly novel strategy to rescue mitochondrial damage and dysfunction in PH, much work remains to establish the efficacy, mechanism, and safety of this approach before clinical translation.

#### 3.3.3 Mitochondrial Autophagy

Mitochondrial autophagy, also known as mitophagy, is essential for maintaining mitochondrial and cellular homeostasis. It helps to clear damaged organelles and cytotoxic protein aggregates to maintain cell homeostasis [105]. A recent study has reported autophagy to be involved in the development of PH. Especially in HPH, selective degradation of mitochondria by mitophagy regulates mitochondrial functions in many cells [106].

FUN14 domain-containing protein 1(FUNDC1) is a hypoxia-induced mitophagy receptor. A recent study employed the hypoxic mitophagy receptor FUNDC1 KO and transgenic (TG) mouse models with hypoxic PH models, and found the upregulation of FUNDC1-induced PASMC proliferation, pulmonary vascular remodeling, and PH. The main mechanisms were found to be a hypoxia-induced increase of FUNDC1 activity which mediates HIF1α activity and PASMC proliferation through increasing ROS production. However, in this experiment, inhibition of mitophagy did not ameliorate RV hypertrophy, which may be because their gain-of- and loss-of-function mitophagy models were not specifically targeted on PASMCs. Nevertheless, in addition to FUNDC1, there are several other mitophagy receptors such as BCL2 and adenovirus E1B 19-kDa-interacting protein 3(BNIP3), BNIP3-like (NIX), and BCL2-like 13 (BCL2L13). Thus, they could also be a therapeutic target for HPH and RV remodeling [107].

#### 3.3.4 Emerging Technology

The present era is witnessing rapid advancements in investigating novel therapeutic applications. We are now looking forward to searching for more specific targets with better efficacy and fewer side effects.

The properties of nanomaterials make them biocompatible, highly targeted, and low toxicity. The therapeutic aim can be reached by changing the magnetic, biochemical, and electronic properties of the nanodrug [108]. Mitochondria are one of the more popular subcellular targets in nanomaterial-associated therapy. Thus far, the development of mitochondrial therapeutics largely focuses on anti-cancer treatment by inducing mitochondria-targeted apoptosis. By using photodynamic therapy/photothermal therapy (PDT/PTT) together with multimodal theranostic imaging, we can monitor and understand the administration of nanotherapeutics and disease progression in a comprehensive manner [109]. Recently, there have been findings about the application of nanomedicine in PH therapy. Compared to traditional medicine, they can prolong the half-life of the drug, improve the stability of the drug, and specifically target the diseased lung tissue to reduce the side effects of the drug [108]. However, we have found few discoveries about mitochondria nanotherapeutics for PH-induced RV remodeling. We believe that mitochondria-targeted nanotherapeutics are still innovative and promising emerging technologies for treating this kind of disease.

Currently, strategies that allow efficient gene editing of mitochondrial genomes are few [109, 110]. The CRISPR/Cas9 technology has been a game-changer in the world of gene editing, opening up new horizons for gene therapy. If we can reliably get nucleic acids into mammalian mitochondria and combine this with the molecular biology tools we already have, we could create a powerful CRISPR/Cas9 gene-editing toolkit for mitochondria. This would be a huge breakthrough because it would allow us to fix genetic defects in mitochondria more accurately, potentially treating many diseases related to mitochondrial dysfunction. In simpler terms, CRISPR/Cas9 gives us more options and greater potential in gene editing [111].

## 4. Conclusions and Perspectives

PH is a disease characterized by increased pulmonary vascular resistance and pulmonary vascular remodeling that can lead to ventricular remodeling. For a long time, treatments related to PAH have focused on dilating the pulmonary vasculature and lowering pulmonary arterial pressure in order to improve patient survival quality [112]. However, even nowadays, when treatments are constantly being updated, PH is not completely curable [113]. In fact, right heart failure is the leading cause of death in patients with PH in the later stages of life [114, 115]. Therefore, the assessment of right heart function and the molecular mechanisms of ventricular remodeling seem to be particularly important in the treatment of PH.

Mitochondria are vital sites for cellular energy metabolism and are involved in biological processes such as signaling, oxidative stress, and apoptosis. In recent years, a large number of studies and data from bioinformatics analyses have all suggested that mitochondrial dysfunction may be an important target in the process of RV remodeling in PAH. One important metabolic characteristic of PAHs has been identified as the Warburg effect, which is the transition from OXPHOS to glycolysis [116, 117, 118]. Furthermore, the balance of mitochondrial dynamics is crucial for maintaining cardiomyocyte integrity, and an imbalance causes not only increased ROS and decreased RV function, but also apoptosis and abnormal proliferation.

The study of mitochondria-targeted medicines has opened up new avenues for PH treatment. Mitochondrial metabolism has been identified as a pivotal target, with strategies aimed at restoring the balance between glycolysis and OXPHOS showing promise. Drugs such as DCA [72], FAO inhibitors [51], and mTOR inhibitors [58] have demonstrated the potential to modulate metabolic pathways, reduce glycolysis, and restore OXPHOS, thereby mitigating RV remodeling and improving pulmonary vascular function.

The exploration of traditional Chinese medicine, such as QLQX, has provided additional insights into the mitigation of RV remodeling through the modulation of mitochondrial-associated apoptotic pathways. The integration of these herbal remedies with modern therapeutics presents a unique opportunity to enhance treatment outcomes in PH [81].

Mitochondrial dynamics, particularly the balance between fission and fusion, have been implicated in the pathophysiology of PH. Inhibitors of Drp1, a key regulator of mitochondrial fission, have shown preclinical efficacy in reversing PH by attenuating mitochondrial dysfunction and cellular proliferation. The development of Drp1 inhibitors like Mdivi-1 [91] and Drp1i27 [93] offers a novel approach to PH treatment, although their clinical translation requires rigorous evaluation.

Potential therapeutic targets and emerging technologies, including mitochondrial transplantation [103], mitochondria-targeted nanotherapeutics [108], and gene editing with CRISPR/Cas9 [112], offer innovative avenues for addressing mitochondrial dysfunction in PH. While these approaches are in their infancy, they hold the promise of targeted therapies that could revolutionize the management of PH and RV remodeling. Since PH is currently incurable, cardiac-specific target therapy might have a significant role in prolonging patient survival. Deletion of the MCJ prevents the development of PH. Reintroduction of MCJ in MCJ KO mice specifically induced right heart hypertrophy without structural changes in the pulmonary vessel. This finding provides new therapeutic ideas for the treatment of pulmonary hypertension [25].

However, there is still a long way to go before these discoveries are translated into therapeutic practice. On the one hand, the efficacy of most investigations is still in the preclinical stage and has yet to be shown in clinical trials. On the other hand, the RV differs significantly from the LV in terms of embryonic development, shape, and hemodynamics, resulting in a distinct response to pharmacological therapy [119]. Drug applications, however, frequently lack relative specificity at this point. Future research should concentrate on establishing the precise molecular pathways behind mitochondrial dysfunction in PH and determining the appropriate therapeutic window for mitochondria-targeted medicines. Furthermore, a thorough assessment of these medications’ long-term safety and effectiveness is also required.
